# ForestPMPlot: A Flexible Tool for Visualizing Heterogeneity Between Studies in Meta-analysis

**DOI:** 10.1534/g3.116.029439

**Published:** 2016-05-18

**Authors:** Eun Yong Kang, Yurang Park, Xiao Li, Ayellet V. Segrè, Buhm Han, Eleazar Eskin

**Affiliations:** * Department of Computer Science, University of California, Los Angeles, California 90095; † Department of Biomedical Informatics and; ‡ Department of Convergence Medicine, University of Ulsan College of Medicine & Asan Institute for Life Sciences, Asan Medical Center, Seoul, 05505 Republic of Korea, 05; § Department of Convergence Medicine, Asan Medical Center, University of Ulsan College of Medicine, Seoul, Republic of Korea; ** Department of Human Genetics, University of California, Los Angeles, California 90095

**Keywords:** GWAS, genetic association studies, heterogeneity, meta-analysis

## Abstract

Meta-analysis has become a popular tool for genetic association studies to combine different genetic studies. A key challenge in meta-analysis is heterogeneity, or the differences in effect sizes between studies. Heterogeneity complicates the interpretation of meta-analyses. In this paper, we describe ForestPMPlot, a flexible visualization tool for analyzing studies included in a meta-analysis. The main feature of the tool is visualizing the differences in the effect sizes of the studies to understand why the studies exhibit heterogeneity for a particular phenotype and locus pair under different conditions. We show the application of this tool to interpret a meta-analysis of 17 mouse studies, and to interpret a multi-tissue eQTL study.

Meta-analysis has become a popular tool for genetic association studies to achieve higher power in identifying genetic variants that affect a trait (Evangelou and Ioannidis 2013). Recently, by combining multiple studies through meta-analysis, a large number of genetic studies have successfully identified novel associated loci that were not identified by any single study included in the meta-analysis (Morris *et al.* 2012; Lango Allen *et al.* 2010; Lambert *et al.* 2013; Heid *et al.* 2010; Anttila *et al.* 2013). As more genetic studies of phenotypes become available, meta-analysis will become even more widely utilized in genetic association studies.

Interpreting and understanding the results of meta-analysis is now becoming important, yet it remains a challenge. The combined studies in a meta-analysis are often heterogeneous. For example, genetic association studies can differ from each other in terms of environmental conditions (Kang *et al.* 2014), study design, populations, statistical noise, and the use of covariates in the analysis (Manning *et al.* 2012). These factors can make the effect sizes differ between studies, a phenomenon called between-study heterogeneity (Han and Eskin 2011). A correct interpretation of this heterogeneity will lead us to a better understanding of the effect under specific conditions, and to an informed decision in the replication study.

In this paper, we describe ForestPMPlot, a flexible visualization tool for analyzing studies included in a meta-analysis. The main feature of the tool is visualizing the differences in the effect sizes of the studies to understand why the studies exhibit heterogeneity for a particular phenotype and locus pair under different conditions. Unlike traditional forest plots, which only display effect size magnitude and its standard error for each study, ForestPMPlot displays the *P*-value and the posterior probability prediction for the existence of the effect in each study (m-value), which is estimated by utilizing cross-study information (Han and Eskin 2012). The main advantage of the m-value is that it can effectively segregate from one another the studies predicted to have an effect, the studies predicted to not have an effect, and the ambiguous studies that are underpowered. ForestPMPlot visualizes the relationship between *P*-values and m-values in a plot called PM-Plot and displays it along with the forest plot. By visualizing much richer information than the traditional forest plot, ForestPMPlot can considerably facilitate the interpretation of the results of meta-analysis.

## Methods

In this section, we first review the two different meta-analysis approaches [fixed effects (FE) model and random effects (RE) model], and explain our approaches for visualizing the results of meta-analysis.

### FE model meta-analysis

The underlying assumption of FE meta-analysis is that the effect size is the same across the studies included in the meta-analysis (Mantel and Haenszel 1959). Under this assumption, in the FE meta-analysis, the effect size estimates of studies, such as the log odds ratios or regression coefficients, are combined and summarized into one summary statistic. The common method of combining effect sizes under FE models is the inverse variance weighted effect size estimate (de Bakker *et al.* 2008; Fleiss 1993). Let $X_{1},\ldots ,X_{c}$ be the effect size estimates from *c* studies included in a meta-analysis and let $V_{i}$ be the variance of $X_{i}$. Then, for FE meta-analysis, the weight for each effect size is set to the inverse variance of the effect size estimate ($W_{i}=V_{i}^{-1}$). Thus the inverse-variance-weighted effect size estimate is

$$\bar{X}=\frac{\sum W_{i}X_{i}}{\sum W_{i}}$$

And the standard error of $\bar{X}$ is ${\sqrt{\sum \;W_{i}}}^{-1}$. Because $\bar{X}$ asymptotically follows a normal distribution, we can compute the FE meta-analysis statistic in the following way.

$$Z_{FE}=\frac{\bar{X}}{{\sqrt{\sum W_{i}}}^{-1}}=\frac{\sum W_{i}X_{i}}{\sqrt{\sum W_{i}}}$$

The above statistic ($Z_{FE}$) follows the standard normal distribution under the null hypothesis of no association. Thus, the *P*-value can be computed by

$$P_{FE}=2\Phi \left(-\left|Z_{FE}\right|\right)$$

where Φ denotes the cumulative density function of the standard normal distribution.

### RE model meta-analysis

Unlike FE model meta-analysis, RE model meta-analysis treats the underlying effect size of each study as a random variable. Specifically, a typical assumption is that the effect size of each study follows a normal distribution with the grand mean $\bar{\beta }$ and the variance $\tau^{2}$ (Han and Eskin 2011; DerSimonian and Laird 1986):

$$\beta_{i}∼N\left(\bar{\beta },\tau^{2}\right).$$

In this model, $\tau^{2}$ represents the between-study variance. In other words, the more the effect sizes of studies included in a meta-analysis differ, the larger the between-study variance ($\tau^{2}$).

Given the above model for effect sizes of the studies, the traditional RE model tests the null hypothesis $\bar{\beta }=0$ *vs.* the alternative hypothesis $\bar{\beta }\neq 0$. Recently, Han and Eskin (2011) showed that, under the condition that there is no heterogeneity under the null hypothesis, which is often the case in genetic association studies, the traditional RE model can be overly conservative. Instead, they proposed a new RE model approach that increases power by testing the null hypothesis $\bar{\beta }=0\text{ and }\tau^{2}=0$ *vs.* the alternative hypothesis $\bar{\beta }\neq 0\text{ or }\tau^{2}\neq 0$. The Han-Eskin model uses the following likelihood model:

$$\begin{array}{l} L_{0}=\prod_{i}\frac{1}{\sqrt{2\pi \sigma_{i}^{2}}}\text{exp}\left(-\frac{\beta_{i}^{2}}{2\sigma_{i}^{2}}\right)\\ L_{1}=\prod_{i}\frac{1}{\sqrt{2\pi \left(\sigma_{i}^{2}+\tau^{2}\right)}}\text{exp}\left(-\frac{{\left(\beta_{i}-\mu \right)}^{2}}{2\left(\sigma_{i}^{2}+\tau^{2}\right)}\right). \end{array}$$

The maximum likelihood estimates $\hat{\mu }$ and ${\hat{\tau }}^{2}$ can be found by an iterative procedure suggested by Hardy and Thompson (1996). Then, the likelihood ratio test statistic can be built

$$S_{Han-Eskin}=\sum \;\text{log}\left(\frac{\sigma_{i}^{2}}{\sigma_{i}^{2}+{\hat{\tau }}^{2}}\right)+\sum \frac{\beta_{i}^{2}}{\sigma_{i}^{2}}-\sum \frac{{\left(\beta_{i}-\hat{\mu }\right)}^{2}}{\sigma_{i}^{2}+{\hat{\tau }}^{2}},$$ (1)

which asymptotically follows a mixture of 1 and 2 degrees of freedom $\chi^{2}$. Accurate *P*-values with small sample correction can be calculated efficiently using precomputed tabulated values (Han and Eskin 2011).

### Identifying studies with an effect through m-value

To distinguish studies with an effect from studies without an effect, we utilize the m-value framework. The m-value (Han and Eskin 2012; Kang *et al.* 2014) is the posterior probability that the effect exists in each study. Thus one can interpret m-value in the following way: a small m-value (*e.g.*, 0.1) represents a study that is predicted to not have an effect, a large m-value (*e.g.*, 0.9) represents a study that is predicted to have an effect, and otherwise it is ambiguous to make a prediction.

In the following, we explain how to compute m-value. Suppose we have *n* studies we want to combine. Let $E=\left[\delta_{1},\delta_{2},\ldots ,\delta_{n}\right]$ be the vector of estimated effect sizes, and $V=\left[V_{1},V_{2},\ldots ,V_{n}\right]$ be the vector of variances of *n* effect sizes. We assume that the effect size $\delta_{i}$ follows the following normal distribution.

$$P\left(\delta_{i}|\text{no}\;\text{effect}\right)=N\left(\delta_{i};0,V_{i}\right)$$ (2)

$$P\left(\delta_{i}|\text{effect}\right)=N\left(\delta_{i};\mu ,V_{i}\right)$$ (3)

We assume that the prior for the effect size is

$$\mu ∼N\left(0,\sigma^{2}\right)$$ (4)

A possible choice for *σ* in genome-wide association studies (GWAS) is 0.2 for small effect and 0.4 for large effects (Stephens and Balding 2009). Let $C_{i}$ be a random variable whose value is 1 if a study *i* has an effect, and 0 otherwise. Let *C* be a vector of $C_{i}$ for *n* studies. Since *C* includes *n* binary variables, *C* can have $2^{n}$ possible configurations. Let $U=\left[c_{1},\ldots ,c_{2^{n}}\right]$ be a vector containing all these possible configurations. We define m-value $m_{i}$ as the probability $P\left(C_{i}=1|E\right)$, which is the probability of study *i* having an effect given the observed effect size estimates. We can compute this probability using the Bayes’ theorem in the following way.

$$m_{i}=P\left(C_{i}=1|E\right)=\frac{\sum_{c\in U_{i}}P\left(E|C=c\right)P\left(C=c\right)}{\sum_{c\in U}P\left(E|C=c\right)P\left(C=c\right)}$$ (5)

where $U_{i}$ is a subset of *U* whose elements’ $i^{th}$ value is 1. Now we need to compute P(E|C=c) and P(C=c). P(C=c) can be computed as

$$P\left(C=c\right)=\frac{B\left(\left|c\right|+\alpha ,n-\left|c\right|+\beta \right)}{B\left(\alpha ,\beta \right)}$$ (6)

where |c| denotes the number of 1s in c, and B denotes the beta function where we set *α* and *β* be 1 (Han and Eskin 2012). The probability *E* given configuration *c*, P(E|C=c), can be computed as

$$P\left(E|C=c\right)=\int_{-\infty }^{\infty }\prod_{i\in c_{0}}N\left(\delta_{i};0,V_{i}\right)\prod_{i\in c_{1}}N\left(\delta_{i};\mu ,V_{i}\right)p\left(\mu \right)\;\text{d}\mu$$ (7)

$$=\bar{C}N\left(\bar{\delta };0,\bar{V}+\sigma^{2}\right)\prod_{i\in c_{0}}N\left(\delta_{i};0,V_{i}\right)$$ (8)

$$\bar{\delta }=\frac{\sum_{i}W_{i}\delta_{i}}{\sum_{i}W_{i}}\;\;\text{and }\;\;\bar{V}=\frac{1}{\sum_{i}W_{i}}$$ (9)

where $c_{0}$ represents the indices of 0 in *c* and $c_{1}$ the indices of 1 in *c*, N(δ;a,b) denotes the probability density function of the normal distribution with mean *a* and variance *b*. $W_{i}=V_{i}^{-1}$ is the inverse variance or precision, and $\bar{C}$ is a scaling factor.

$$\bar{C}=\frac{1}{{\left(\sqrt{2\pi }\right)}^{N-1}}\sqrt{\frac{\prod_{i}W_{i}}{\sum_{i}W_{i}}}exp\left\{-\frac{1}{2}\left(\sum_{i}\;W_{i}\delta_{i}^{2}-\frac{{\left(\sum_{i}W_{i}\delta_{i}\right)}^{2}}{\sum_{i}W_{i}}\right)\right\}$$ (10)

### PM-plot

**Figure 1:**
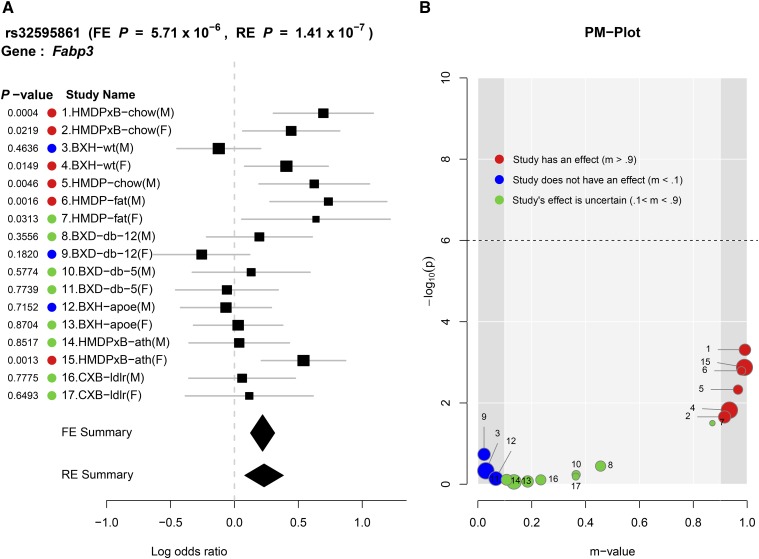
Seventeen mouse HDL studies with various environmental/genetic conditions are combined in this meta-analysis (Kang *et al.* 2014). In this example, we want to focus on three BXH-wt(M) and four BXH-wt(F) studies. These BXH strains are F2 mice constructed from a cross between C57BL/6J × C3H/HeJ F2 wild-type strains under western diet conditions (van Nas *et al.* 2010), but differing by sex. When we consider the effect size estimates only in forest plot format, two confidence intervals of effect estimates overlap each other, making it ambiguous if the observed heterogeneity is a result of stochastic errors. However, in the PM-Plot, since the m-values are calculated utilizing cross-study information, the posterior probabilities are well segregated for these two studies (m-value: 0.93 *vs.* 0.03), allowing us to hypothesize that the SNP effects on HDL in C57BL/6J × C3H/HeJ F2 strains under the western diet condition can be interacting with sex. Implicated genes are *Fabp3*, also known as fatty acid binding protein 3, which is a well-known gene playing a regulatory role at the nexus of lipid metabolism and signaling including HDL-cholesterol, LDL-cholesterol, and fasting insulin (Mitchell *et al.* 1996; Zhang *et al.* 2013). (A) Forest plot and (B) PM-plot for rs32595861 locus (*Fabp3* gene) analyzing data from the Kang *et al.* (2014) study. FE, fixed effects model; RE, random effects model.

**Figure 2:**
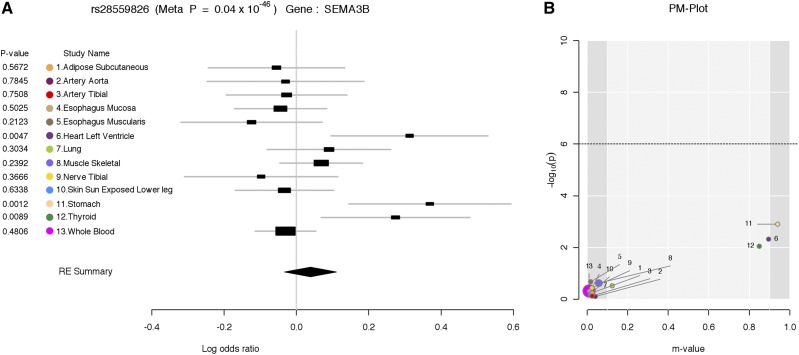
Thirteen multiple-tissue eQTL studies analyzed in GTEx Consortium (2015). In this example, 13 different tissue eQTLs were analyzed together for SEMA3B gene expression levels. The first column shows the *P*-value for each tissue specific eQTL study. The different colored dots represent the different tissues, the study name column shows the various tissue names included in this multi-tissue eQTL analysis. The forest plot shows that the SNP rs28559826 shows a better association with the *SEMA3B* gene expression level in three tissues (heart left ventricle, stomach, and thyroid), although the confidence intervals overlap between many tissues. On the other hand, the PM-plot clearly shows that association of the top three tissues (heart left ventricle, stomach, and thyroid) are outstanding compared to other tissue eQTLs. The gene *SEMA3B* is also known as the semaphorin/collapsin family of molecules. This gene plays a critical role in the guidance of growth cones during neuronal development. It has been shown to act as a tumor suppressor by inducing apoptosis (SEMA3B 2015). (A) Forest plot and (B) PM-plot for rs28559826 locus (*SEMA3B* gene) analyzing data from the GTex study (GTEx Consortium 2015). RE, random effects model.

For interpreting and understanding the result of a meta-analysis, it is informative to look at the *P*-values and m-values at the same time. We propose the PM-plot framework (Han and Eskin 2012), which plots the *P*-values and m-values of each study together, and visualizes the relationship between m-values and *P*-values in a two-dimensional space. Through the PM-Plot, a researcher can easily distinguish which study is predicted to have an effect, and which study is predicted not to have an effect. The *x*-axis of the PM-Plot represents the m-value between 0 and 1, the *y*-axis represents the statistical significance of association, $-log_{10}$(*P*-value), and the dashed horizontal line is the significance threshold. The colored circle for each study is placed in the PM-Plot according to its m-value and *P*-value. We classify the estimated posterior probability for each study into three categories: a study that has an effect (m ≥ 0.9) is denoted by a red dot, a study that does not have an effect (m ≤ 0.1) is denoted by a blue dot, and a study whose effect is uncertain (0.1 < m < 0.9) is denoted by a green dot. The dot size represents the study’s sample size. Figure 1B shows one example of a PM-plot. One reason that studies are ambiguous (0.1 < m < 0.9) is that they are underpowered due to small sample size. If the sample size increases, the study can be drawn to either the left or the right side. ForestPMPlot utilizes an automatic algorithm to place the study names (numbers corresponding to the actual names in the forest plot) to minimize the overlap between labels (Lemon 2006). For the multiple tissue eQTL application (Figure 2), we can add particular color for dots that represents the corresponding tissue type.

### Data availability

The authors state that all data necessary for confirming the conclusions presented in the article are represented fully within the article.

## Results and Discussion

### Application to GWAS meta-analysis

Figure 1 is an example of the output of ForestPMPlot for a mouse HDL study (Kang *et al.* 2014), which combines 17 mouse studies. The 17 HDL mouse studies included in this meta-analysis have different environmental/genetic conditions, such as diet (high fat/low fat, etc.) and various gene knockouts, including homozygous deficiency in leptin receptor (db/db), LDL receptor knockouts, and *Apoe* gene knockouts. In Figure 1, study names describe the characteristic of each study. In this example, the study name is encoded as {mouse-strain}-{condition} format. HMDP stands for hybrid mouse diversity panel, which combines classic inbred strains for mapping resolution, and recombinant inbred strains for mapping power (Bennett *et al.* 2010). Mice for the HMDPxB panel were created by breeding females of the various HMDP inbred strains (Bennett *et al.* 2010) to males carrying transgenes for both Apoe Leiden (van den Maagdenberg *et al.* 1993) and for human Cholesterol Ester Transfer Protein (CETP) (Jiang *et al.* 1992) on a C57BL/6 genetic background, which cause the progression of atherosclerosis along the arterial tree. BXH wild type (BXH/wt) mice were produced as previously described (van Nas *et al.* 2010). Briefly, C57BL/6J mice were intercrossed with C3H/HeJ mice to generate 321 F2 progeny. BXD-db strain is an F2 intercross between the inbred strains DBA/2 and C57BL/6 (Davis *et al.* 2012). The male C57BL/6 parents carried heterozygosity deficiency in the leptin receptor (db +/−), and F1 progeny were selected for homozygosity of the mutant allele. Among F2 progeny, only those with homozygous deficiency in leptin receptor (db/db) were selected. For CXB-ldlr strain, female BALB/cByJ-LDLRKO (designated as C) mice were crossed with male C57BL/6J-LDLRKO (designated as B) to generate F1 mice. Then, an intercross of F1 was performed to generate F2 mice.

Given the heterogeneous natures of studies that differ in many different dimensions, interpreting a significant but heterogeneous result can be challenging. Examining both the forest plot and the PM-Plot allows us to generate an appropriate hypothesis on why the effect size differences are occurring. For example, consider studies 3 and 4, which contain mouse C57BL/6J × C3H/HeJ F2 wild-type strains under the western diet condition (van Nas *et al.* 2010), but differ by sex. These two studies show heterogeneous effects in the forest plot, but the two confidence intervals of effect estimates overlap each other, making it ambiguous if the observed heterogeneity is a result of stochastic errors. A researcher can perceive, however, that there exist other studies that have similar effect sizes to these two studies, increasing our belief that this observed heterogeneity is driven by true interactions of sex, genetic background, and diet. Nevertheless, the forest plot alone does not display or systematically infer such cross-study information. In the PM-Plot, the m-values are calculated utilizing cross-study information. The posterior probabilities are well segregated for these two studies (m-value: 0.93 *vs.* 0.03), allowing us to hypothesize that the SNP effects on HDL in C57BL/6J × C3H/HeJ F2 strains under the western diet condition can be interacting with sex. This shows an example where our visualization framework can lead to plausible interpretations, which would not have been straightforward had we used the traditional forest plot alone.

### Application to multi-tissue eQTL analysis

One powerful application of our proposed framework is in multi-tissue eQTL analysis in the Genotype-Tissue Expression (GTEx) project. The GTEx project studies human gene expression and genetic regulation in multiple tissues, providing valuable insights into the mechanisms of gene regulation, which can lead to the new discovery of disease-related perturbations. In this project, genetic variation between individuals will be examined for correlation with differences in gene expression level to identify regions of the genome that influence whether, and by how much, a gene is expressed. In particular, examining multiple tissues can give us valuable insights into the genetic architecture of the regulatory mechanism, because many regulatory regions are known to act in a tissue-specific manner (Ernst *et al.* 2011; Encode Project Consortium 2012). Hence, understanding the role of regulatory variants, and the tissues in which they act, is essential for the functional interpretation of GWAS loci and insights into disease etiology.

Figure 2 is an example of the output of ForestPMPlot for a multi-tissue eQTL study for SEMA3B gene (GTEx Consortium 2015). Examining both the forest plot and the PM-Plot allows us to obtain an insight into the tissue-specific genetics effects in eQTL analysis, which leads to the identification of three significant eQTL tissues (heart left ventricle, stomach, and thyroid). This example clearly shows that examining both the forest plot and the PM-Plot allows us to easily hypothesize that there is a specific group of studies showing tissue differences in eQTL analysis.

### Conclusions

In conclusion, we describe ForestPMPlot, a flexible visualization tool for analyzing studies included in a meta-analysis, such as meta-analysis of GWAS. The main feature of the tool is visualizing the differences in the effect sizes of studies for better understanding of why the studies exhibit heterogeneity. Unlike the traditional forest plot framework, which displays only effect size magnitude and its standard error for each study, ForestPMPlot additionally displays the posterior probability prediction for the existence of the effect in each study, and the *P*-values. This allows us to effectively segregate from one another studies predicted to have an effect, and studies predicted not to have an effect. Through visualization of these estimates and predictions, ForestPMPlot can considerably facilitate the interpretation of the results of meta-analysis. We show an example analysis where our visualization framework leads to plausible interpretations of gene-by-environment interaction and multiple tissue eQTL, which would not have been straightforward with the traditional framework.
